# Supplementary figures and images for: Eukaryotic CD-NTase, STING, and viperin proteins evolved via domain shuffling, horizontal transfer, and ancient inheritance from prokaryotes
Source: PLoS Biol. 2023 Dec 8;21(12):e3002436. doi: 10.1371/journal.pbio.3002436 (PMC10732462; doi:10.1371/journal.pbio.3002436)

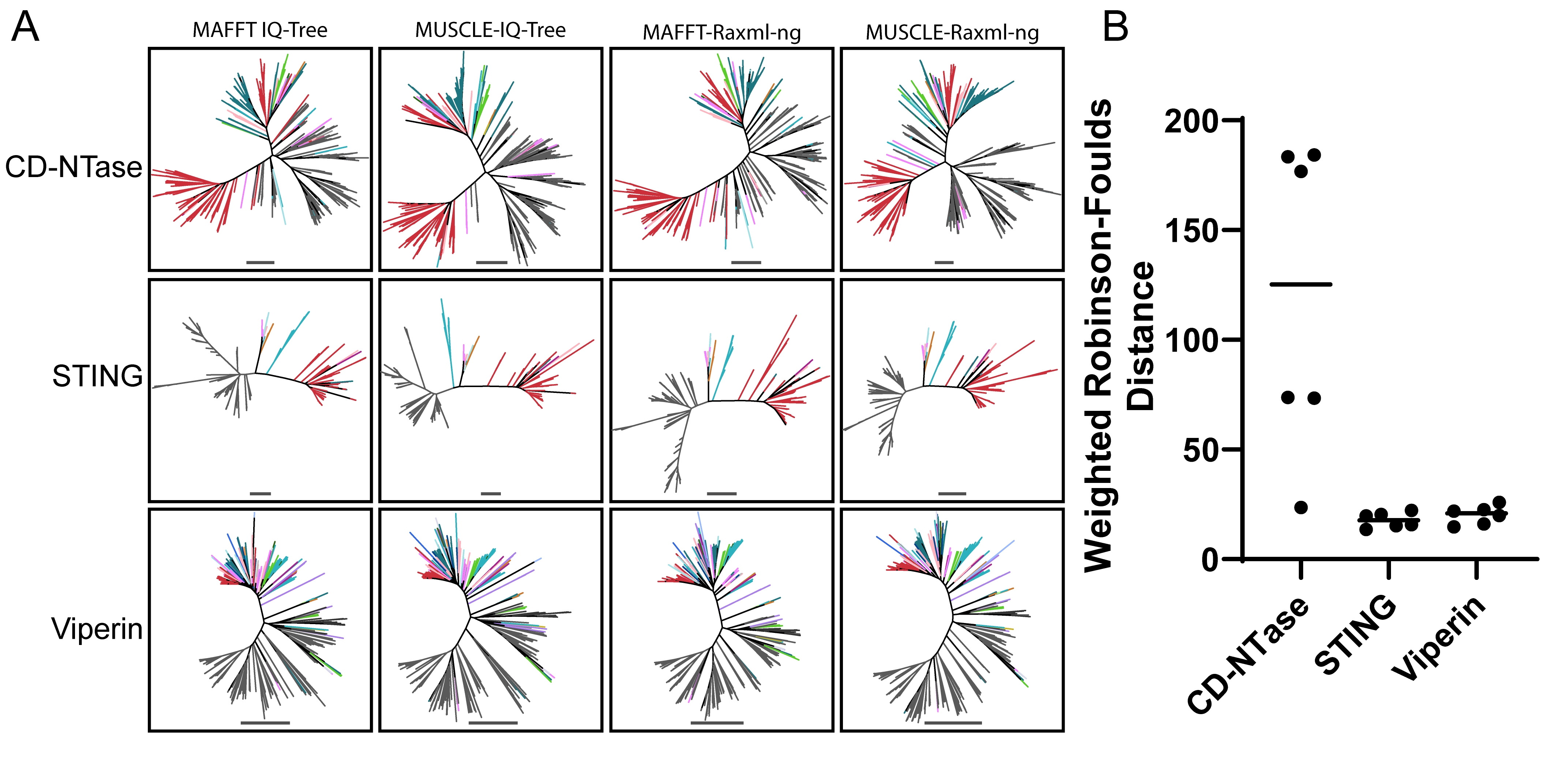

Supplement: S3 Fig — (A) Unrooted maximum likelihood phylogenetic trees generated from 2 separate alignments (MUSCLE and MAFFT) and with 2 different tree inference programs (IQtree and RaxML-ng). Scale bar of 1 shown beneath each tree represents the number of amino acid substitutions per position in the underlying alignment. Colored branches show eukaryotic sequences with the same color scheme as Fig 1B, while gray lines are bacterial sequences. For the majority of relationships discussed here, we recovered the same tree topology at key nodes regardless of alignment or tree reconstruction algorithm used. (B) The weighted Robinson–Foulds distances all pairwise comparisons between the 4 tree types (MAFFT/MUSCLE alignment built with IQTREE/RAXML-ng). Although the distances were higher for the CD-NTase tree (as expected for this highly diverse gene family), all of the key nodes defining the cGLR, OAS, and eSMODS superfamilies, as well as their nearest bacterial relatives, were well supported (>70 ultrafast bootstrap value). Underlying alignment and Newick files are included (Alignments: S9, S10, S11, S12, S13, S14 Files. Newick files: S2, S4, S8, S15, S16, S17, S18, S19, S20, S21, S22, S23 Files) under Supporting information. All pairwise comparisons for weighted Robinson–Foulds distance calculations are included in S1 File. (TIF) [file pbio.3002436.s003.tif]

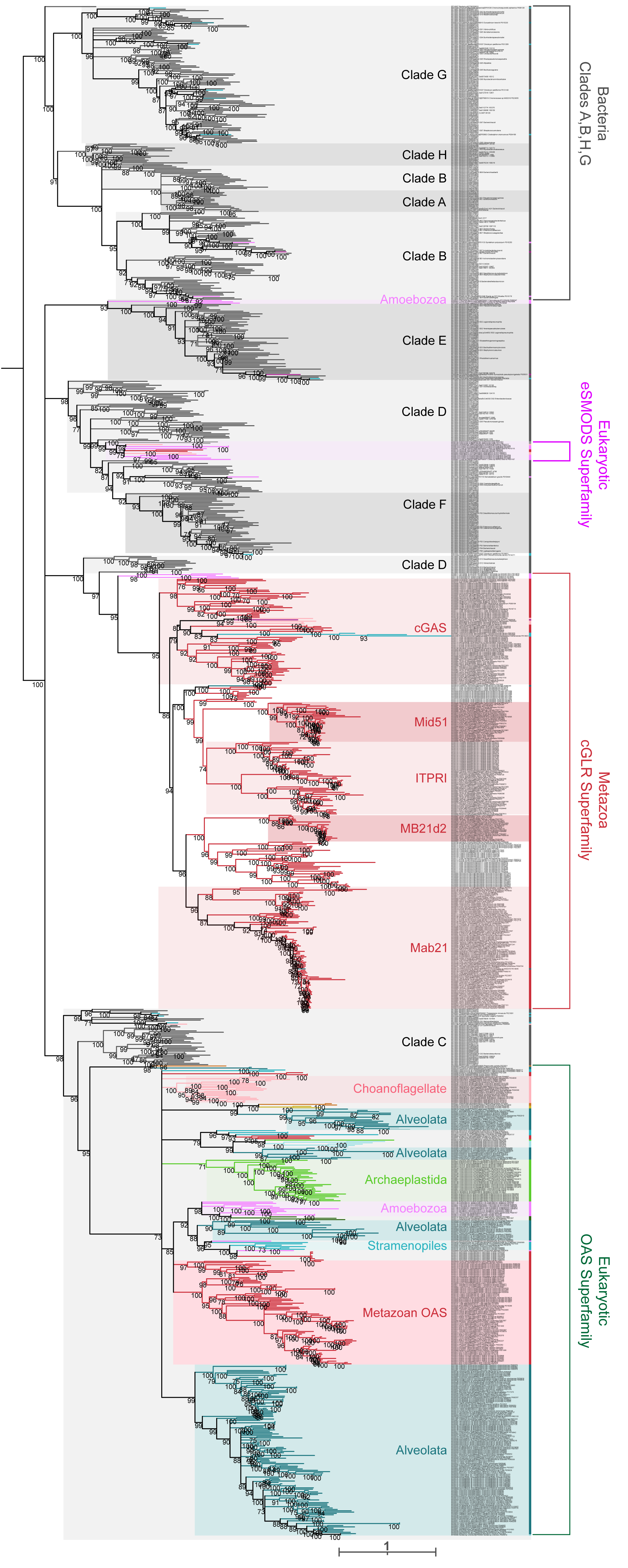

Supplement: S4 Fig — Maximum likelihood phylogenetic tree generated by IQtree of hits from iterative HMM searches for diverse eukaryotic CD-NTases. Tree is arbitrarily rooted between bacterial CD-NTase clades. Scale bar represents the number of amino acid substitutions per position in the underlying MUSCLE alignment. Eukaryotic sequences are color coded as in Fig 1B. Ultrafast bootstrap values calculated by IQtree at all nodes with support >70 are shown. Branches with support values <70 were collapsed to polytomies. Underlying Newick file is included in S2 File under Supporting information. (TIF) [file pbio.3002436.s004.tif]

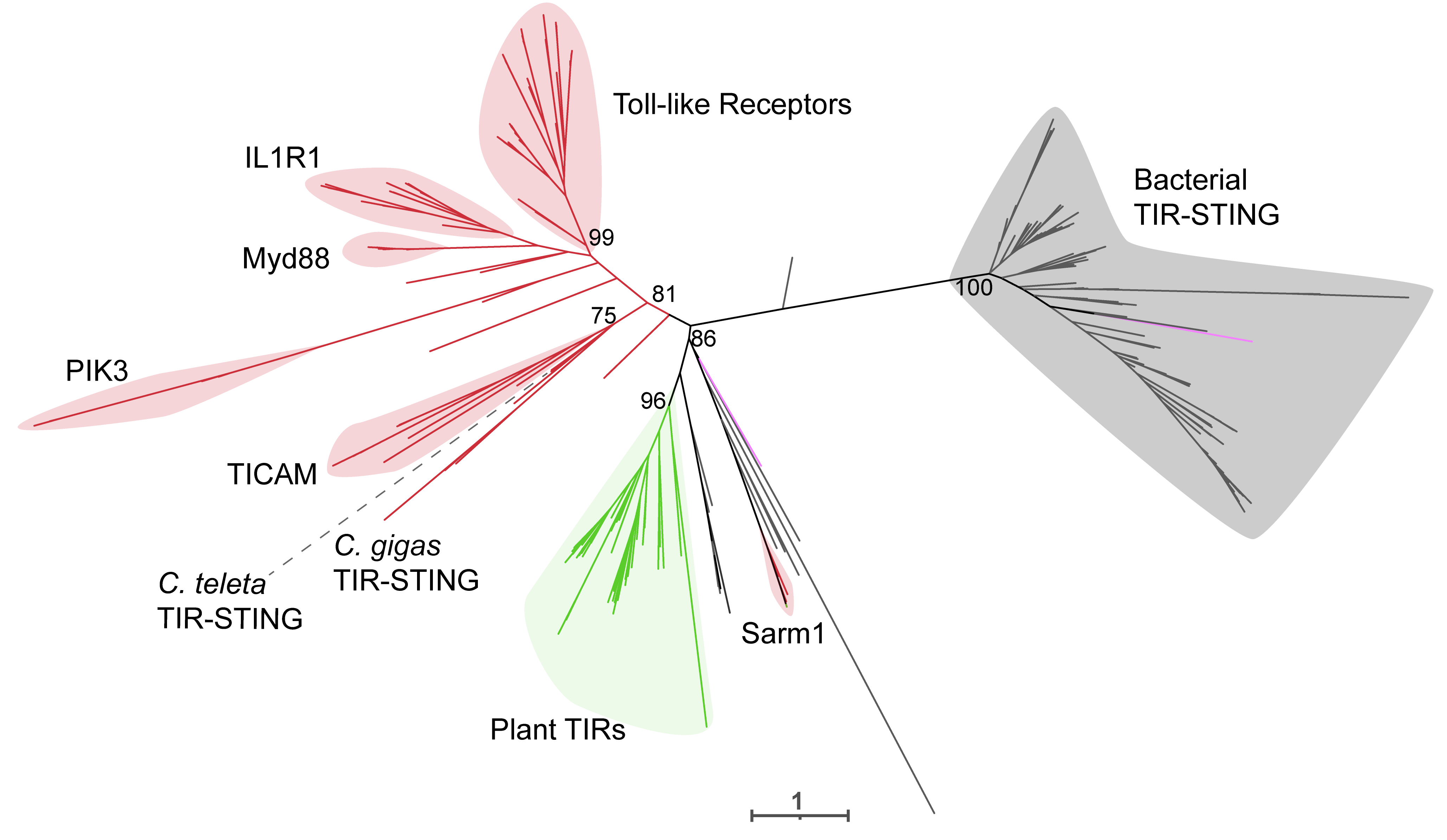

Supplement: S7 Fig — Unrooted maximum likelihood tree of diverse TIR domains. Scale bars on the phylogenetic tree represent the number of amino acid substitutions per position in the underlying MUSCLE alignment. Eukaryotic sequences are color coded as in Fig 1B. Ultrafast bootstrap values calculated by IQtree at key nodes are shown. Underlying Newick file is included in S24 File under Supporting information. (TIF) [file pbio.3002436.s007.tif]
