# Supplementary material for: Eukaryotic CD-NTase, STING, and viperin proteins evolved via domain shuffling, horizontal transfer, and ancient inheritance from prokaryotes
Source: PLoS Biol. 2023 Dec 8;21(12):e3002436. doi: 10.1371/journal.pbio.3002436 (PMC10732462; doi:10.1371/journal.pbio.3002436)
Supplement: Attachment — Submitted filename: 139799_1_rebuttal_2840996_s3mm1l.docx [file pbio.3002436.s040.docx]

**Manuscript number:** RC-2023-02160

**Corresponding author(s):** Tera Levin

*[Please use this template only if the submitted manuscript should be considered by the affiliate journal as a full revision in response to the points raised by the reviewers.*

*If you wish to submit a preliminary revision with a revision plan, please use our "*[*Revision Plan*](https://docs.google.com/document/u/0/d/1HHp62Tfeb44nd3Gu_3ChB0b8gmMdEog7GjZcZFwHSiQ/edit)*" template.* ***It is important to use the appropriate template to clearly inform the editors of your intentions.****]*

## 1. General Statements [optional]

*Through Review Commons, we received some highly favorable and constructive feedback from reviewers who are clearly knowledgeable about phylogenomics and/or the field of bacterial anti-phage immunity. We have responded to all suggestions made by the reviewers, which we feel have substantially improved and clarified the manuscript. We thank all three reviewers for their thoughtfulness and time.*

## 2. Point-by-point description of the revisions

**Reviewer #1**

**Evidence, reproducibility and clarity**

Culbertson and Levin present an elegant computational analysis of the evolutionary history of several families of immune proteins conserved in bacteria and metazoan cells. The authors' work is impressive, revealing interesting insight into previously known connections and identifying exciting new connections that further link bacterial anti-phage defense and animal innate immunity. The results are overall well-presented and will have an important impact on multiple related fields. I have a few comments for the authors to help explain some of the new connections observed in their findings and clarify the results for a general audience.

*We thank the reviewer for their kind appraisal of our manuscript as well as their helpful comments. We found their comments to be very useful in strengthening our work and increasing the clarity of the writing.*

 Comments:
 1) The authors adeptly navigate difficult and changing nomenclature around cGAS-STING signaling but there may be room for clarifying terminology. Although historically the term "CD-NTase" has been used to describe both bacterial and animal enzymes (including by this reviewer's older work as well), the field has now settled on consistent use of the name "CD-NTase" to describe bacterial cGAS/DncV-like enzymes and the use of the names "cGAS" and "cGLR" to describe animal cGAS-like receptor proteins. Nearly all papers describing bacterial signaling use the term CD-NTase, and since 2021 most papers describing divergent cGAS-like enzymes in animal signaling now use the term "cGLR" (for recent examples see primary papers Holleufer et al 2021 PMID 34261128; Slavik et al 2021 PMID 34261127; Li et al 2023 PMID 37379839; Cai et al 2023 PMID 37659413 and review articles Cai et al 2022 PMID 35149240; Slavik et al 2023 PMID 37380187; Fan et al 2021 PMID 34697297; West et al 2021 PMID 34373639 Unterholzner Cell 2023 PMID 37478819). Kingdom-specific uses of CD-NTase and cGLR may help add clarity to the manuscript especially as each group of enzyme is quite divergent and many protein members synthesize signaling molecules that are distinct from cyclic GMP-AMP (i.e. not cGAS).

 Related to this point, the term "SMODS" is useful for describing the protein family domain originally identified in the elegant work of Burroughs and Aaravind (Burroughs et al 2015 PMID 26590262), but this term is rarely used in papers focused on the biology of these systems. "eSMODS" is a good name, but the authors may want to consider a different description to better fit with current terminology.

*We appreciate the reviewer’s suggestion and have updated the text to try to be more clear (ex: using cGLR as a more specific term whenever possible). However, as OAS is distinctly not a cGLR, strict kingdom-specific use of the terms CD-NTase and cGLR is not possible. We have updated the Mab21 superfamily to be re-named as the cGLR superfamily, as those seem to be synonymous based on recent literature. At this time we are choosing to stick with the eSMODS terminology as it remains to be shown that these eukaryotic proteins have a CD-NTase-like biochemical function.*

*An example of how we have tried to navigate this naming issues is:*

*“The cGLR superfamily passed all four of these HGT thresholds, as did another eukaryotic clade of CD-NTases that were all previously undescribed. We name this clade the eukaryotic SMODS (eSMODS) superfamily, because the top scoring domain from hmmscan for each sequence in this superfamily was the SMODS domain (PF18144), which is typically found only in bacterial CD-NTases (Supplementary Data).”*

2) The authors state that proteins were identified using an iterative HMM-based search until they "began finding proteins outside of the family of interest" (Line 86). Is it possible to please explain in more detail what this means? A key part of the analysis pipeline is knowing when to stop, especially as some proteins like CD-NTases and cGLRs share related-homology to other major enzyme groups like pol-beta NTases while other proteins like STING and viperin are more unique.

*We have updated the text to better explain how we determined that a given protein sequence was excluded:*

*“After using this approach to create pan-eukaryotic HMMs for each protein family, we then added in bacterial homologs to generate universal HMMs (Fig. 1A and Supp. Fig. 1), continuing our iterative searches until we either failed to find any new protein sequences or began finding proteins outside of the family of interest (Supp. Fig. 1). To define the boundaries that separated our proteins of interest from neighboring gene families, we focused on including homologs that shared protein domains that defined that family (see Materials and Methods for domain designations) and were closer to in-group sequences than the outgroup sequences on a phylogenetic tree (outgroup sequences are noted in the Materials and Methods). “*

*We also added a section to the Methods specifically defining our outgroups:*

*“As outgroup sequences, we used Poly(A) RNA polymerase (PAP) sequences for the CD-NTases, and molybdenum cofactor biosynthetic enzyme (MoaA) for viperin. We did not have a suitable outgroup for STING domains, nor did any diverged outgroups come up in our searches.”*

3) The authors comment on several controls to guard against potential contaminating bacterial sequences present in metazoan genome sequencing datasets (Lines 174-182). It may be helpful to include this very important part of the analysis as part of the stepwise schematic in Figure 1a. Additionally, have the authors used other eukaryotic features like the presence of introns or kingdom specific translation elements (e.g. Shine-Dalgarno- vs. Kozak-like sequences) as part of the analysis?

*We agree that it will be very interesting to look for these eukaryotic gene features, both to rule out contamination and to discern how eukaryotes have acquired and domesticated bacteria-like immune proteins. However, one limitation when working with the data in EukProt is that many species are represented by de novo transcriptome datasets and therefore information about the local gene environment, introns, or promoters are unavailable.*

 4) A particularly surprising result of the analysis is a proposed connection between oligoadenylate synthase-like (OAS-like) enzymes and bacterial Clade C CD-NTases. A concern with these results is that previous structural analysis has demonstrated that bacterial CD-NTase enzymes and animal cGLRs are more closely related to each other than they are to OAS (Slavik et al 2021 PMID 34261127). Can the authors provide further support for a connection between OAS and Clade C CD-NTases? The C-terminal alpha-helix bundle of OAS is known to be distinct (Lohöfener et al 2015 PMID 25892109) and perhaps AlphaFold2 modeling of bacterial Clade C CD-NTases and additional OAS sequences may provide further bioinformatic evidence to support the authors' conclusions.

*We were also surprised by this finding as it seems to be in opposition to structural comparisons in studies such as Whiteley et. al  2019 (PMID 30787435). As the reviewer suggests,e used AlphaFold to predict the structures of two CD-NTases, that of Bacterioides uniformis (Clade C016) and Escherichia coli (Clade C018) as well as a previously uncharacterized OAS-like protein (Tripos fusus P058904) and compared those structural predictions to those of cGAS (PDB: 6CTA), OAS1 (PDB: 4RWO), and DncV (PDB: 4TY0). We used the DALI server to make these all vs all comparisons.*

*We have not included these analyses in the manuscript as the results were largely inconclusive. The average pairwise z-score between any of these structures was around 20, with a narrow range of scores between 16 (e.g. OAS vs. DncV) and 22 (e.g. DncV vs. the Clade C CD-NTases). For reference, the z-score of a given protein compared to itself was ~50 and a z-score of 20 is a general DALI benchmark used to determine if structures are homologous ( z-scores between 8-20 are in a gray area, and 20+ are generally considered homologous).*

*In our view, these pairwise structural comparisons suffer from essentially the same problem that is evident in phylogenetic trees containing only animal and bacterial homologs. Namely, all structures/sequences under consideration are extremely different from each other, on very long branches that are difficult to place with confidence when few homologs are being considered. The benefit of our approach is that we have the ideal species diversity to break up the long branches (particularly with respect to the OAS superfamily), allowing us to place those sequences confidently on the phylogeny.*

*That said, while we have strong support for the topology of OAS within the CD-NTase tree, the interpretation of the relationships relies partly on the inferred root of the tree. In our analyses, we opted not to include a distant outgroup such as pol-beta for rooting purposes, as these sequences aligned poorly with the CD-NTases, resulting in a substantial decrease in alignment and tree quality. Instead, in Fig. 2 we present a tree that is arbitrarily rooted within the bacterial CD-NTases, as this root allows for clade C to be phylogenetically coherent. Our data are also consistent with an alternative rooting, placing OAS as an outgroup. If so, this would yield a tree that implies that OAS-like sequences could have given rise to all other CD-NTases and that, within the non-OAS sequences, all bacterial CD-NTases emerged from within Clade C. We thought it slightly more likely that the root of CD-NTases was solidly within bacteria, hence the display we chose. However, we were not intending to rule out an OAS-outgroup model here. As this response to reviewers will be publically available alongside the final manuscript, we hope this clarifies our claims about the placement of OAS.*

.

5) One of the most exciting results in the paper is identification of a family of putative CD-NTase enzymes conserved in metazoans. Although full description may be beyond the scope of this paper, if possible, some more analysis would be interesting here:
 a. Are these CD-NTase enzymes in a conserved gene neighborhood within the metazoan genomes (i.e. located next to a potential cyclic nucleotide receptor?)
 b. Do these metazoan genomes encode other known receptors for cyclic nucleotide signaling (PFAM searches for CARF or SAVED domains for instance).
 c. Similar to points 3 and 4, is it possible to add further evidence for support of these proteins as true metazoan sequences that have predicted structural homology to bacterial CD-NTase enzymes?

*Yes agreed, we think point a is an exciting avenue of questioning to pursue. However, as mentioned above, the Eukprot dataset often does not provide the relevant information for the analyses proposed. Therefore, we feel that answering questions about the genomic region of these proteins is beyond the scope of the current manuscript. In particular, all 6 of the eSMODS species are represented only by transcriptomes, making these analyses impossible.*

*For point b, we searched EukProt with HMMs for SAVED domains (PF18145), finding 24 total SAVED-containing proteins in EukProt. (We did not find a CARF HMM in Pfam, Tigrfam or other databases, and so could not easily carry out these searches.) Five of the 24 SAVED-containing sequences came from species encoding an eSMODS gene. This represented 3 species out of the total 20 species where we detected a SAVED domain. While this is a potentially intriguing overlap, we cannot make a strong claim about whether these SAVED sequences derive from eukaryotes vs. bacterial contamination without undergoing the extensive searching and phylogenetic tree construction methods for SAVED domains that we have performed for our three families of interest. We expect this will be an interesting line of inquiry for a future study.*

*For point c, we agree that additional evidence to support the finding that the eSMODS are eukaryotic rather than bacterial sequences would be helpful. To us, the strongest pieces of evidence would be: 1) presence of eukaryotic gene architecture, 2) adjacency to clearly eukaryotic genes in the contig, and/or 3) fluorescence in situ hybridization experiments in these species to localize where the genes are encoded. Unfortunately, the transcriptome data available does not provide this level of information. We hope that other groups will follow up on these genes and species to decide the matter more definitively. In the meantime, we feel that our filters for HGT vs. contamination have done as much as possible with the existing dataset. We have modified the text in this region to leave open potential scenarios that could be fooling us, such as the presence of unusual, long-term, eukaryote-associated symbionts in the taxa where we detect eSMODS:*

*“For species represented only by transcriptomes, these criteria may still have difficulty distinguishing eukaryote-bacteria HGT from certain specific scenarios such as the long-term presence of dedicated, eukaryote-associated, bacterial symbionts. However, because these criteria allow us to focus on relatively old HGT events, they give us higher confidence these events are likely to be real. ”*

6) The authors state that obvious CD-NTase/cGLR enzymes are not present in organisms that encode the group of divergent eukaryotic "blSTINGs". Have the authors analyzed the protein-coding genes encoded immediately upstream and downstream of the blSTING proteins with AlphaFold2 and FoldSeek? It would be very exciting if putative cyclic nucleotide generating enzymes are predicted to be encoded within the nearby gene neighborhood.

*Similar to the eSMODS, the majority of the species with blSTINGs were represented by transcriptomes (22/26). We do agree that this type of analysis would be very interesting. However, we feel that this is beyond the scope of this manuscript.*

7) Line 144 appears to reference the incorrect supplementary figure. SI Figure 4 may be the correct reference?

*We agree and have made this change. We thank the reviewer for catching this error.*

I hope the authors will find my comments useful, thank you for the opportunity to read this exciting manuscript.

**Significance**

Culbertson and Levin present an elegant computational analysis of the evolutionary history of several families of immune proteins conserved in bacteria and metazoan cells. The authors' work is impressive, revealing interesting insight into previously known connections and identifying exciting new connections that further link bacterial anti-phage defense and animal innate immunity. The results are overall well-presented and will have an important impact on multiple related fields. I have a few comments for the authors to help explain some of the new connections observed in their findings and clarify the results for a general audience.

**Reviewer #2**

**Evidence, reproducibility and clarity**

Describe your expertise? Molecular Evolution, Mechanisms of Protein evolution, Phylogenomics, Adaptation.

 Summary: This manuscript broadly aims to improve our understanding the evolutionary relationships between eukaryote and bacterial protein families where members of those families have immune roles. The study focuses on three such families and samples deeply across the eukaryotic tree. The approaches taken include a nice application of the EukProt database and the use of homology detection approaches that are sensitive to the issues of assigning homology through deep time. The main findings show the heterogeneity in means by which these families have arisen, with some of the families originating at least as far back as the LCA of eukaryotes, in contrast the wide spread yet patchy distribution of other families is the result of repeated independent HGT events and/or convergent domain shuffling.

*We thank the reviewer for this excellent review and their helpful comments and suggestions. We firmly believe that these comments will strengthen and clarify our work.*

 Major Comments:
 1. Overall the level of detail provided throughout the manuscript is lacking, perhaps the authors were constrained by a word limit for initial submission, if so then this limit needs to be extended to include the detail necessary. In addition, there are some structural issues throughout, e.g. some of the very brief intro (see later comment) reads a little more like methods (paragraph 2) and abstract (paragraph 3). The results section is lacking detail of the supporting evidence from the clever analyses that were clearly performed and the statistics underpinning conclusions are not included.

*Good suggestion, we have updated the paper to include more details and statistics on the analyses that were performed. We have also expanded on some of the most interesting findings about these bacterial innate immune proteins in the introduction (see Comment 2 below for our changes), as well as shifting the methods-like paragraph mentioned (paragraph 2) to later on in the paper. For paragraph 3, we have slimmed this down to include fewer details, but leave the final paragraph of the Introduction as a brief synopsis to prime the reader for the rest of the paper.*

2. The intro and discussion both include statements about some recent discoveries that bacteria and mammals share mechanisms of innate immunity - but there is no further detail into what would appear to be important work leading to this study. This context needs to be provided in more detail therefore I would encourage the authors to expand on the intro to include specific detail on these significant prior studies. In addition, more background information on the gene families investigated in detail here would be useful e.g. how the proteins produced influence immunity etc should be a feature of the intro. A clear and concise rationale for why these 3 particular gene families (out of all the possible innate immune genes known) were selected for analysis.

*We have added in additional background about some of the most exciting discoveries made in the past few years. We also included specific rationale as to why we chose to look at cGAS, STING, and Viperin.*

*Specifically, we have added the following to the introduction:*

*“ For example, bacterial cGAS-DncV-like nucleotidyltransferases (CD-NTases), which generate cyclic nucleotide messengers (similar to cGAS), are massively diverse with over 6,000 CD-NTase proteins discovered to date. Beyond the cyclic GMP-AMP signals produced by animal cGAS proteins, bacterial CD-NTases are capable of producing a wide array of nucleotide signals including cyclic dinucleotides, cyclic trinucleotides, and linear oligonucleotides* [*[11,14]*](https://paperpile.com/c/BTEck9/rG0I+fKiu)*. Many of these bacterial CD-NTase products are critical for bacterial defense against viral infection*[*[8]*](https://paperpile.com/c/BTEck9/Rls8)*. Interestingly, these discoveries with the CD-NTases mirror what has been discovered with bacterial viperins. In mammals, viperin proteins restrict viral replication by generating  3’-deoxy-3’,4’didehdro- (ddh) nucleotides*[*[4,15–17]*](https://paperpile.com/c/BTEck9/2zA3P+xFWJr+AYjG+PmpTX) *block RNA synthesis and thereby inhibit viral replication*[*[15,18]*](https://paperpile.com/c/BTEck9/2zA3P+sxFkL)*. Mammalian viperin generates ddhCTP molecules while bacterial viperins can generate ddhCTP, ddhUTP, and ddhGTP. In some cases, a single bacterial protein is capable of synthesizing two or three of these ddh derivatives*[*[4]*](https://paperpile.com/c/BTEck9/AYjG)*. These discoveries have been surprising and exciting, as they imply that some cellular defenses have deep commonalities spanning across the entire Tree of Life, with additional new mechanisms of immunity waiting to be discovered within diverse microbial lineages. But despite significant homology, these bacterial and animal immune proteins are often distinct in their molecular functions and operate within dramatically different signaling pathways (reviewed here*[*[5]*](https://paperpile.com/c/BTEck9/N7ooJ)*). How, then, have animals and other eukaryotes acquired these immune proteins?”*

*In regards to why we choose to investigate CD-NTases, STING, and Viperin specifically, we have added the following to the third paragraph of the introduction:*

*“We choose to focus on the cGAS, STING, and Viperin for a number of reasons. First, in metazoans cGAS and STING are part of the same signaling pathway whereas bacterial CD-NTases often act independently of bacterial STINGs[21], raising interesting questions about how eukaryotic immune proteins have gained their signaling partners. Also, given the vast breadth of bacterial CD-NTase diversity, we were curious as to if any eukaryotes had acquired CD-NTases distinct from cGAS. For similar reasons, we investigated Viperin, which also has a wide diversity in bacteria but a much more narrow described function in eukaryotes.”*

3. Context: Genome quality is always a concern, and confirming the absence of an element/protein in a genome is challenging given the variation in quality of available genomes. Low BUSCO scores mean that the assessment of gene loss is difficult to evaluate (but we are not provided with said scores). Query: in the results section it states that the BUSCO completeness scores (which need to be provided) etc were insufficient to explain the pattern of gene loss. I would like to know how they reached this conclusion - what statistical analyses (ANOVA?? OTHER??) have been performed to support this statement and please include the associated P values etc. Similarly, throughout the paper, including in the discussion section, the point is brushed over. If, given a statistical test, you find that some of the disparity in gene presence is explained by BUSCO score, most of your findings are still valid. It would just be difficult to make conclusions about gene loss.

*We have rewritten this section to be more clear about what we feel we can and cannot say about gene loss and BUSCO scores. This section now reads:*

*“However, outside of Metazoa, these homologs were sparsely distributed, such that for most species in our dataset (711/993), we did not recover proteins from any of the three immune families examined (white space, lack of colored bars, Fig. 1B). While some of these absences may be due to technical errors or dataset incompleteness (Supp. Fig. 2), we interpret this pattern as a reflection of ongoing, repeated gene losses across eukaryotes, as has been found for other innate immune proteins*[*[27–29]*](https://paperpile.com/c/BTEck9/CkD0i+JrWwD+DZcXY) *and other types of gene families surveyed across eukaryotes*[*[28,30–32]*](https://paperpile.com/c/BTEck9/zAkST+dPLT9+dYyK8+JrWwD)*. Indeed, many of the species that lacked any of the immune homologs were represented by high-quality datasets (Ex: Metazoa, Chlorplastida, and Fungi). Thus, although it is always possible that our approach has missed some homologs, we believe the resulting data represents a fair assessment of the diversity across eukaryotes, at least for those species currently included within EukProt.”*

*In addition, we direct readers to EukProt v3, where the BUSCO scores are publicly available.*

*“BUSCO scores can also be viewed on EukProt v3 (https://evocellbio.com/SAGdb/images/EukProtv3.busco.output.txt).”*

4. In terms of the homolog search strategy - line 394 - can you please state what an "outgroup gene family" means in this context. It is unclear but very important to the downstream interpretation of results.

*We have updated the materials and methods to specifically name our outgroups:*

*“As outgroup sequences, we used Poly(A) RNA polymerase (PAP) sequences for the CD-NTases, and molybdenum cofactor biosynthetic enzyme (MoaA) for viperin. We did not have a suitable outgroup for STING domains, nor did any diverged outgroups come up in our searches.”*

 5. For reproducibility, the materials and methods section needs to provide more detail/sufficient detail to reproduce these results. E.g the section describing phase 1 of the euk searches the text here repeats what is in the results section for the crystal structure work but doesn't give me any information on how, what method was used to "align the crystal structures", what scoring scheme is used and how the scoring scheme identifies "the core"? What specific parameters are used throughout. Why is MAFFT the method of choice for some of the analyses? Whereas, in other cases both MAFFT and MUSCLE are employed. What are the specific settings used for the MAFFT alignments throughout - is it default (must state if that is the case) or is it MAFFT L-INS-I with default settings etc.

*We have updated the text to include the specific settings used each time a particular software package was deployed. We also have included information for STING as to how we aligned 3 published crystal structures to determine the boundaries of homology.*

*Here is how we now discuss identifying the “core” STING domain:*

*“ For STING, where the Pfam profile includes regions of the protein outside of the STING domain, we generated a new HMM for the initial search. First, we aligned crystal structures of HsSTING (6NT5), Flavobacteriaceae sp. STING (6WT4) and Crassostrea gigas STING (6WT7) with the RCSB PDB “Pairwise Structure Alignment” tool with a jFATCAT (rigid) option*[*[73,74]*](https://paperpile.com/c/BTEck9/Bogh+xMzi)*. We  defined a core “STING” domain, as the ungapped region of 6NT5 that aligned with 6WT7 and 6WT4 (residues G152-V329 of 6NT5).Then we aligned 15 eukaryotic sequences from PF15009 (all 15 of the “Reviewed” sequences on InterPro) with MAFFT(v7.4.71)*[*[75]*](https://paperpile.com/c/BTEck9/y2dOV) *with default parameters and manually trimmed the sequences down to the boundaries defined by our crystal alignment (residues 145-353 of 6NT5). We then trimmed the alignment with TrimAI (v1.2)*[*[76]*](https://paperpile.com/c/BTEck9/iwLR5) *with options -gt 0.2. The trimmed MSA was then used to generate an HMM profile with hmmbuild from the hmmer (v3.2.1) package (hmmer.org) using default settings. “*

*We employed three alignment softwares at specific times throughout our analyses. MAFFT was used as our default aligner for most of the analysis. Hmmalign (part of the hmmer package) was used to make the alignments prior to hmmbuild. The overall goal of this work was to reconstruct the evolutionary history of these proteins via a phylogenetic tree. To ensure that this tree topology was as robust as possible we employed the more computationally intensive, but more accurate, tree builder MUSCLE. We have updated the text in the methods section to be more clear as to why we used each software.*

*We have updated the methods section to read:*

*“MUSCLE was deployed in parallel with MAFFT to generate these final alignments to ensure that the final tree topology would be as robust as possible. MUSCLE is a slightly more accurate but more computationally intensive alignment software*[*[79]*](https://paperpile.com/c/BTEck9/WGeg)*.”*

6. The justification for the number of HMM searches needs to be included. The choice of starting points for the HMMs was cryptic - please provide details. It is likely that you ran the search until no more sequences were found or until sequences were added from a different gene family, and that these happened to be between 3 and 5 searches, but it reads like you wanted to run it 3 or 5 times and that corresponds to the above condition. Something like this would be clearer: "The profile was [...] until no more sequences were found or until sequences from other gene families were found which was between 3 and 5 times in all cases" - the same is true of figure 1.

*We agree that this could have been worded better. We have updated the text to make it more clear that we searched until saturation which happened to occur between 3-5 searches and not that we arbitrarily wanted to do 3-5 searches.*

*We have updated the text, which now reads:*

*“After using this approach to create pan-eukaryotic HMMs for each protein family, we then added in bacterial homologs to generate universal HMMs (Fig. 1A and Supp. Fig. 1), continuing our iterative searches until we either failed to find any new protein sequences or began finding proteins outside of the family of interest (Supp. Fig. 1). To define the boundaries that separated our proteins of interest from neighboring gene families, we focused on including homologs that shared protein domains that defined that family (see Materials and Methods for domain designations) and were closer to in-group sequences than the outgroup sequences on a phylogenetic tree (outgroup sequences are noted in the Materials and Methods). “*

*We also updated the figure legend to Fig. 1. It now reads:*

*“Each set of searches was repeated until few or no additional eukaryotic sequences were recovered which was between 3-5 times in all cases.”*

7. Why do you limit hits to 10 per species - might this lead to misleading findings about gene family diversity? Info and justification for approach is required (411-412).

*We limited the hits to 10 per species to limit the influence of any one species on our alignments and subsequent phylogenetic trees. This 10-per-species cap was never reached with any search for STING or Viperin, but was used to throttle the number of Metazoan hits when searching for CD-NTases. Because of this, we probably have missed some amount of the diversity of Metazoan Mab21-like/OAS-like sequences, although this was not a focus of our manuscript. We have updated the text to be more clear about why we have included this limit and when the limit was invoked.*

*We have update the text, which now reads:*

*“HMM profiles were used to search EukProt via hmmsearch (also from hmmer v3.2.1) with a statistical cutoff value of 1e-3 and -hit parameter set to 10 (i.e. the contribution of a single species to the output list is capped at 10 sequences). It was necessary to cap the output list, as EukProt v3 includes de novo transcriptome assemblies with multiple splice isoforms of the same gene and we wanted to limit the overall influence a single species had on the overall tree. We never reached the 10 species cap for any search for STING or viperin homologs; only for the CD-NTases within Metazoa did this search cap limit hits.”*

8. The information in Supplementary Figure 3 is quite difficult to assess visually, but I think that is what is expected from that figure. However, this is an important underpinning element of the work and should really be quantitatively assessed. A metric of comparison of trees, with defined thresholds etc there are many out there, even a simple Robinson-Foulds test perhaps? Essentially - comparing the panels in Supplementary Figure 3 by eye is unreliable and in this case not possible given there are no labels. It would also be important to provide these full set of phylogenies generated and associated RF/other scores as supplementary file.

*We agree that this Supplementary Figure is difficult to assess by eye, however we feel that it is vital to show this data. Visually, we do feel like this figure conveys the idea that while individual branches may move around, the major clades/areas of interest are stable across the different alignments and tree builders. To increase robustness, we have included the weighted Robinson-Foulds test results into a new panel of this figure (Supplementary Fig. 3B).*

*We have added a section to the methods on how this weighted Robinson-Foulds test was conducted:*

*“Weighted Robinson-Foulds distances for Supp. Fig. 3B were calculated with Visual TreeCmp (settings: -RFWeighted -Prune trees -include summary -zero weights allowed)*[*[83]*](https://paperpile.com/c/BTEck9/GDGt)*.”*

*We added the weighted Robinson-Foulds data to Supplemental Fig. 3 and have updated the figure legend to reflect this new data. The new legend for Supp. Fig. 3B reads:*

*“(B) The average weighted Robinson-Foulds distances all pairwise comparisons between the four tree types (MAFFT/MUSCLE alignment built with IQTREE/RAXML-ng). Although the distances were higher for the CD-NTase tree (as expected for this highly diverse gene family), all of the key nodes defining the cGLR, OAS, and eSMODS superfamilies, as well as their nearest bacterial relatives, were well supported (>70 ultrafast bootstrap value).”*

9. Does domain shuffling mean that phylogenetic reconstruction is less valid? How was the alignment performed in these cases to account for this.

*Thank you for bringing this up, this is a point we have now clarified in the text. Our searches, alignments, and trees are all of single protein domains, as typically only conservation within domains is retained across the vast distances between bacteria and eukaryotes. As such, domain shuffling should have no impact on the validity of that phylogenetic reconstruction. We have updated the text to be more clear about the scope of the alignments and searches. We made changes to our wording throughout the manuscript. One specific example of this is:*

*“Using maximum likelihood phylogenetic reconstruction on the STING domain alone, we identified STING-like sequences from 26 diverse microeukaryotes whose STING domains clustered in between bacterial and metazoan sequences, breaking up the long branch.”*

 Minor Comments:
 10. I am not sure about the use of the term "truly ancestral" or variants thereof, same issues with "significant homology" and "inherited since LECA and possibly longer" .. these are awkwardly phrased. E.g. I think perhaps "homologous across the whole length" might be clearer, and elsewhere "present in LECA and possibly earlier" may be more fitting.

*We have updated the text for these phrases throughout the manuscript and have replaced them with more specific language.*

11. Line 75 - "Detecting" rather than discovering?

*We appreciate the suggestion. However, because many of these gene families have never been described in the eukaryotic lineages considered here, we think ‘discovering’ is more appropriate. Indeed, the eSMODS lineage demonstrates that our search approach has the power to find not just new homologs but to discover totally new subfamilies of these eukaryotic proteins.*

12. 132-133 - more justification is needed for the choice of bacterial genes.

*We have clarified that our selection of bacterial CD-NTases included every known CD-NTase at the time of our analysis. The text now reads:*

*“As representative bacterial CD-NTases, we used 6,132 bacterial sequences, representing a wide swath of CD-NTase diversity*[*[43]*](https://paperpile.com/c/6RKR01/hCyUs)*. To our knowledge, this dataset included every known bacterial CD-NTase at the time of our analysis.”*

13. For the downsizing from 6000 to 500 what were the criteria and thresholds.

*We have updated the text to include the PDA software options for downsampling.The text now reads:*

*“We downsampled the CD-NTase bacterial sequences from ~6000 down to 500 using PDA software (options -k 500) on a FastTree (default settings) tree built upon a MAFFT (default parameters) tree, to facilitate more manageable computation times on alignments and tree construction.“*

14. How are you rooting your trees e.g. figure 2? Information is provided for Viperin but not others.

*We have updated the text to ensure that the root of every tree is specifically stated.*

15. In the results section on CD-NTases I think it would be best to place the second paragraph detailing the role of cGAS earlier in this section, perhaps after the first sentence.

*We have moved the second paragraph, which introduces cGAS, OAS, and the other CD-NTases to the beginning of the CD-NTase section.The first paragraph of the CD-NTase section of the results now reads:*

*“We next studied the evolution of the innate immune proteins, beginning with cGAS and its broader family of CD-NTase enzymes. Following infections or cellular damage, cGAS binds cytosolic DNA and generates cyclic GMP-AMP (cGAMP)*[*[32–35]*](https://paperpile.com/c/6RKR01/YWaLW+P4DER+9SozD+KU3Ls)*, which then activates downstream immune responses via STING* [*[34,36–38]*](https://paperpile.com/c/6RKR01/z28tE+9SozD+wfAoQ+L4jzJ)*. Another eukaryotic CD-NTase, 2’5’-Oligoadenylate Synthetase 1 (OAS1), synthesizes 2',5'-oligoadenylates which bind and activate Ribonuclease L (RNase L)*[*[39]*](https://paperpile.com/c/6RKR01/Iuyl4)*. Activated RNase L is a potent endoribonuclease that degrades both host and viral RNA species, reducing viral replication (reviewed here*[*[40,41]*](https://paperpile.com/c/6RKR01/SskMx+IlGUP)*). Some bacterial CD-NTases such as DncV behave similar to animal cGAS; they are activated by phage infection and produce cGAMP*[*[8,42,43]*](https://paperpile.com/c/6RKR01/VO72M+hCyUs+wVXvg)*. These CD-NTases are commonly found within cyclic oligonucleotide-based anti-phage signaling systems (CBASS) across many bacterial phyla and archaea*[*[8,27,43]*](https://paperpile.com/c/6RKR01/wVXvg+WMMAE+hCyUs)*.”*

16. Is FASTtree really necessary to include as it will underperform in all instances? Removing that method and comparing the remaining two (i.e. IQTREE and RAXML) - what level of disagreement do you find between the 2 alignment and 2 tree building methods? The cases that disagree should also be detailed.

*We agree that FASTtree underperforms against IQTREE and RAXML and have eliminated those trees from the supplement. We initially had included FASTtree, as it still seems to be widely used in phylogenetic analyses within the recent papers on bacterial immune homologs, but we completely agree with the reviewer and have removed it. In addition, we have calculated and added in the average weighted Robinson-Foulds Distance to Supplemental Figure 3. Our manuscript focuses on features of the phylogenetic trees that were consistent across all the replicate methods. However, given the numerous sequences and high degree of divergence involved, there were many cases where individual branches shifted between the methods, e.g. if individual CD-NTases within bacterial clade G swapped positions with one another. The differences we observed between the trees were inconsequential to our overall conclusions.*

17. Again a structural point - the start to paragraph "To understand the evolutionary history of CD-NTases we used the Pfam domain PF03281 as a starting point", I don't know at this point why or how you have done this. The sentence seems a little premature. I would therefore suggest that you start that paragraph with your motivation, "In order to..." and then finish that paragraph with your sentence in quotes above which actually summarizes the paragraph.

*We have updated the text to clear up this paragraph (in addition to other structural changes in the CD-NTase section. The paragraph containing information about how we started the HMM searches for the CD-NTases now reads:*

*“ To begin our sequence searches for eukaryotic CD-NTases, we used the Pfam domain PF03281, representing the main catalytic domain of cGAS, as a starting point. As representative bacterial CD-NTases, we used 6,132 bacterial sequences, representing a wide swath of CD-NTase diversity*[*[21]*](https://paperpile.com/c/BTEck9/3QErt)*. Following our iterative HMM searches, we recovered 313 sequences from 109 eukaryotes, of which 34 were metazoans (Supplemental Data and Fig. 1B). Within the phylogenetic trees, most eukaryotic sequences clustered into one of two distinct superfamilies: the cGLR superfamily (defined by clade and containing a Mab21 PFAM domain: PF03281) or the OAS superfamily (OAS1-C: PF10421) (Fig. 2A). Bacterial CD-NTases typically had sequences matching the HMM for the Second Messenger Oligonucleotide or Dinucleotide Synthetase domain (SMODS: PF18144).”*

18. Line 148 - "within" change to "before"?

*We have updated the text with this suggestion.*

19. Unclear from text as is whether you found any STING homologs in arthropods (~line 157). Please update the text for clarity. Would also suggest that "agreeing" should be replaced with "aligning".

*We found several STING homologs in arthropods and have updated the text to specifically note this. We also have updated the text as per the suggestion of using the term “aligning” instead of “agreeing”.The text now reads:*

*“Almost half of these species (10/19) were arthropods, aligning with prior findings of STING sparseness among arthropods*[*(Wu et al. 2014)*](https://paperpile.com/c/BTEck9/aEIJ)*. We did find STING homologs in 8/19 arthropod species in EukProt v3, including the previously identified STINGs of Drosophila melanogaster, Apis mellifera and Tribolium castaneum*[*(Wu et al. 2014; Margolis, Wilson, and Vance 2017)*](https://paperpile.com/c/BTEck9/aEIJ+6HaP)*.”*

20. Line 169 - If clade D is not a clade, maybe it should be called something different.

*Yes, unfortunate naming, isn’t it?  Clade D is not a coherent clade in our results nor when it was first described, but we feel that for consistency with the rest of the field, it is best if we adhere to previously published nomenclature.*

21. Line 188-190 - In principle, max likelihood should be able to infer the right tree even with high divergence.

*Yes, we agree that maximum likelihood methods should be able to infer the correct tree. However, we are not sure what change the reviewer is suggesting here.*

22. Paragraph starting at 199 - eSMODS - always unknown function or mostly - could be important.

*To our knowledge the function of the two closest bacterial CD-NTases to the eSMODS group have an unknown function.*

23. For calling HGT you state that one of the criteria is that the euk and bac sequences branched near one another, what is "near" in this scenario?

*“Near” in this case refers to being adjacent on the phylogenetic tree. We have updated the text for clarity. The text now reads:*

*“To minimize such false positive HGT calls, we took a conservative approach in our analyses, considering potential bacteria-eukaryote HGT events to be trustworthy only if: 1) eukaryotic and bacterial sequences branched adjacent to one another with strong support (bootstrap values >70); 2) the eukaryotic sequences formed a distinct subclade, represented by at least 2 species from the same eukaryotic supergroup; 3) the eukaryotic sequences were produced by at least 2 different studies; and 4) the position of the horizontally transferred sequences was robust across all alignment and phylogenetic reconstruction methods used (Supp. Fig. 3A).”*

24. In legends be specific about what type of support value, e.g. bootstrap or jack-knife.. I think it is always bootstrap but would be good to have that precision.

*Our phylogenetic trees only use bootstrap values for support and so have updated the figure legends and methods to provide this information. Apologies for this lack of clarity.*

25. Throughout the text if stating e.g. "clustered robustly and with high support" please provide the appropriate values.

*We have updated the text to provide bootstrap values when invoking statements about support. An example of this is:*

*“There are two clades of Chloroplastida (a group within Archaeplastida) sequences that branch robustly (>80 ultrafast bootstrap value) within the bacteria clade.”*

26. It is unclear from the text how the animal origin of the TIR domain is supported (~line 274). Please provide necessary details to support your statements in the results section.

*Our phylogenetic tree of TIR domains (Supp. Fig. 7), places C. gigas’ TIR domain (of its STING protein) clusters with high support next to other metazoan TIR domains.*

*We have updated the STING section to include these lines:*

*“We also investigated the possibility that C. gigas acquired the TIR-domain of its TIR-STING protein via HGT from bacteria, however this analysis also suggested an animal origin for the TIR domain (Supp. Fig. 7), as the C. gigas TIR domain clustered with other metazoan TIR domains such as Homo sapiens TICAM1 and 2 (ultrafast bootstrap value of 75). Eukaryotic TIR-STINGs are also rare, further supporting the hypothesis that this protein resulted from recent convergence, where animals independently fused STING and TIR domains to make a protein resembling bacterial TIR-STINGs, consistent with previous reports*[*[19]*](https://paperpile.com/c/BTEck9/94aX6)*.”*

27. Replace similar with -> similar "to"

*We have accepted the suggestion and replaced “with” with “to”.*

28. Line 266: It was previously shown .. or it is known but not "it was previously known"

*We have rephrased the sentence to be clearer: “Some eukaryotes like C. gigas…”.*

29. The last sentence in paragraph ~line 277: "Our work also identified a number of non-metazoan STINGS...." Please expand on this and provide some of the details on this finding in the text or point to the figure that supports the statement and provide a little more detail here.

*The intent of the words on line 277 was a summary of what we had previously discussed in the STING section. For clarity we updated the text, which now reads:*

*“Interestingly the non-metazoan, blSTINGs (Fig. 3C) that are found in the Stramenopiles, Haptista, Rhizaria, Choanoflagellates and Amoebozoa have a TM-STING domain architecture similar to animal STINGs but a STING domain more similar to bacterial STINGs..”*

*blSTINGs are discussed in more detail earlier in the STING section (specifically paragraph 3) where we say:*

*“Using maximum likelihood phylogenetic reconstruction on the STING domain alone, we identified STING-like sequences from 26 diverse microeukaryotes whose STING domains clustered in between bacterial and metazoan sequences, breaking up the long branch. We name these sequences the bacteria-like STINGs (blSTINGs) because they were the only eukaryotic group of STINGs with a bacteria-like Prok_STING domain (PF20300) and because of the short branch length (0.86 vs. 1.8) separating them from bacterial STINGs on the tree (Fig. 3C). While a previous study reported STING domains in two eukaryotic species (one in Stramenopiles and one in Haptista)* [*[19]*](https://paperpile.com/c/BTEck9/94aX6)*, we were able to expand this set to additional species and also recover blSTINGs from Amoebozoa, Rhizaria and choanoflagellates. This diversity allowed us to place the sequences on the tree with high confidence (bootstrap value >70), recovering a substantially different tree than previous work*[*[19]*](https://paperpile.com/c/BTEck9/94aX6)*. As for CD-NTases, the tree topology we recovered was robust across multiple different alignment and phylogenetic tree construction algorithms (Supp. Fig. 3A).”*

30. Line 294: it is unclear which are the orphan taxa -we are directed to figure 1 but there is no notation for orphan taxa here perhaps add something to the figure to make obvious which these are.

*We have updated the text to mention these orphan taxa specifically by name.*

*The text now reads:*

*“The 194 viperin-like proteins we recovered came from 158 species spanning the full range of eukaryotic diversity, including organisms from all of the major eukaryotic supergroups, as well as some orphan taxa whose taxonomy remains open to debate (Fig. 1, Ancyromonadida, Hemimastigophora, Malawimonadida).”*

 31. Lines 340-341 - some redundant use of eukaryotic/eukaryotes

*We have updated the text to reduce redundancy.*

32. Lines 475-480 - some further detail needed - how were sequences trimmed to the TIR domain? - what were your starting sequences? etc.

*We have updated the text detailing how we acquired a set of proteins from Interpro and how we used hmmscan to determine the coordinates for the TIR domains in those proteins. We then isolated the TIR domains (using the coordinates defined by hmmscan) and proceeded to align those sequences*

*The text now reads:*

*“We used hmmscan to identify the coordinates of TIR domains in a list of 203 TIR domain containing-sequences from InterPro (all 203 proteins from curated “Reviewed” selection of IPR000157 (Toll/interleukin-1 receptor homology (TIR) domain as of 2023-04-04)) and 104 bacterial TIR-STING proteins (the same TIR-STING proteins used in Fig. 3)*[*[3]*](https://paperpile.com/c/BTEck9/2VPWU)*. Next, we trimmed the sequences down to the hmmscan identified TIR coordinates and aligned the TIR domains with MUSCLE (-super5). We trimmed the alignments with TrimAL and built a phylogenetic tree with IQtree (-s, -bb 1000, -m TEST, -nt AUTO).”*

33. Check that the colour schemes for branches etc are detailed in the legends of supplementary as well as main.

*We have updated the text of figure legends to be more clear about our maintenance of the same color scheme throughout the manuscript. This involved ensuring that the following statement (or an equivalent statement) was present in the figure legends of Figures 2, 3, 4, S2, S3,S4,S5,S6, and S7:*

*“Eukaryotic sequences are colored according to eukaryotic group as in Fig. 1B.”*

34. The threshold set for gaps is very strict at 0.2. This seems quite strict given the sequences are potentially quite highly divergent. What length are the alignments that you are using after trimming - these details need to be included and considered.

*We have updated the text to specifically detail how long our alignments were after trimming and how that post-trimming length compares to the length of the alignment for each PFAM group.*

*Specifically, the text now reads:*

*“The length of these final alignments were 232, 175, and 346 amino acids long for CD-NTases, STING, and viperin respectively. These alignments represent ≥75% of the length of alignment their respective PFAM domain (PF3281 (Mab-21 protein nucleotidyltransferase domain) for CD-NTases, PF20300 (Prokaryotic STING domain) for STING, and PF404055 (Radical SAM family) for viperin.”*

35. How were sequences downsampled with PDA? Line 424.

*We have updated the text to include the PDA settings that were used to downsample sequences. The text now reads:*

*“To ensure the combined HMM did not have an overrepresentation of either bacterial or eukaryotic sequences, we downsampled the bacterial sequences and eukaryotic sequences to obtain 50 phylogenetically diverse sequences of each, and then combined the two downsampled lists. To do this, eukaryotic and bacterial sequences were each separately aligned with MAFFT (default parameters), phylogenetic trees were built with FastTree (v2.1.10)*[*[77]*](https://paperpile.com/c/BTEck9/Cvbdg)*, and the Phylogenetic Diversity Analyzer (pda/1.0.3)*[*[78]*](https://paperpile.com/c/BTEck9/mDw8i) *software with options -k 50 or -k 500 with otherwise default parameters was run the the FastTree files to downsample the sequences while maximizing remaining sequence diversity.”*

36. Please provide adequate descriptions for the materials in the supplementary files for the manuscript, they currently lack description. They are useful and we fully support their inclusion with sufficient information.

*We have expanded the descriptions of the provided supplementary files.*

37. The starting sequences, hmm pipeline and scripts would be great to include, apologies if we have missed them.

*We have added the starting bacterial sequences to the supplementary data, as well as the final HMMs, and the one script that we used in our analysis. All other software (including the included script) is freely and publicly available.*

**Significance**

This study provides us with examples of instances where a medley of different mechanisms have resulted in the emergence of innate immune proteins across eukaryotes. The study is entirely bioinformatic in nature and provides some nice cases for future study. The thorough search strategies are to be commended. The limitations of the work are that we don't know whether the functions have also been conserved across deep time and/or in the independent events described. Nevertheless, this work contributes to a growing body of evidence on the complex, and sometimes shared, nature of the evolution of animal and bacterial immunity. I would classify this nice study as a conceptual advance of our understanding of the evolution of protein families through deep time and would imagine it is of interest to a broad audience of biologists from immunologists to evolutionary biologists and structural biologists.

**Reviewer #3 (Evidence, reproducibility and clarity (Required)):**

 The manuscript by Culbertson and Levin takes a bioinformatic approach to investigate the evolutionary origins/trajectories of three different proteins domains involved in innate immunity in both bacteria and eukaryotes: cGAS/CD-NTases, STING, and Viperins. To perform this analysis, the authors apply an iterative homology search model to the EukProt database of eukaryotic genomes. Their analysis finds that that eukaryotic CD-NTases arose from multiple horizontal gene transfer events between bacteria and eukaryotes. They also fill in an important gap in understanding how STING from bacteria evolved into modern human STING by identifying blasting in diverse eukaryotes. Finally, they determine that Viperins are an ancient protein family that likely existed in LECA, but found two more recent HGT events for proteins related in Vipirin.

 Major comments

 1. The hypothesis for the origin of STING via convergent domain shuffling could be handled with a little more care in the text. The authors show that homologs of STING from animals can also be found in the genomes of diverse eukaryotes outside the metazoa, demonstrating (1) STING and cGAS have had different histories, and (2) that these sequences are more bacteria-like than metazoan STING. However, in multiple places (the title, line 275, elsewhere) the term "convergence" could be misleading. "Convergence" leaves the reader with the impression that there is no common ancestor between the STING domain from bacteria and eukaryotes. I understand that the authors are using "convergent domain shuffling" to draw this distinction, but I'm unsure if a naïve reader will glean the distinction between domain shuffling and STING itself converging. I would argue that we simply cannot place eukaryotic STING and blSTING proteins on the tree of bSTING sequences. i.e. blSTING are no more related to bacterial TM-STING than bacterial TIR-STING (likely the missing bSTING sequences are simply extinct?). Can the authors curate their language to state more simply that STING likely arose through horizontal gene transfer, but it is unlikely that bacterial TM-STING is the unequivocal progenitor?

*We thank the reviewer for this comment, and we absolutely agree that we should be clearer about the distinction between convergence and convergent domain shuffling. We have changed the title and edited the text to increase clarity. In addition, we have clarified what our data does and does say about the evolutionary history of STING. We feel that our STING tree (Fig.3 C), due to a general sparseness of eukaryotic and bacterial sequences, is insufficient to confidently call if eukaryotes acquired STING by HGT or if STING was present in the LECA.*

*We have added the following to clear up this issue:*

*“Overall, the phylogenetic tree we constructed (Fig. 3C) suggests that there is domain-level homology between bacterial and eukaryotic STINGs, but due to sparseness and lack of a suitable outgroup, this tree does not definitively explain the eukaryotic origin of the STING domain. However, the data does clearly support a model in which convergent domain shuffling in eukaryotes and bacteria generated similar TM-STING and TIR-STING proteins independently.”*

Minor Comments

 1. Spelling error in Figure 3B and 3C: "cannoical"

*Thanks, we have corrected this error.*

2. Figure 5 could be improved to more clearly articulate the findings of the manuscript. In A, it's unclear how OAS relates to Mab21 and a reader not paying close attention might think that OAS was part of the gene duplications after Mab21 was acquired. The LECA origins of OAS are also not presented (albeit, these are still defined in the legend). In B, this panel would suggest that there was not horizontal transfer of STING from bacteria to eukaryotes but rather both domains of life received STING from a separate source. My understanding is STING did likely arise in bacteria, however, the assumption that extant TM-STING in bacteria is the predecessor of TM-STING in eukaryotes is not well supported. Similarly for the TIR domain.

*We have updated Fig. 5 to more clearly show that OAS was likely in the LECA and that eSMODS and cGLRs were HGT’d from bacteria to other eukaryotic lineages. For STING, it was not our intent to imply that the extant TM-STING in bacteria is the predecessor of TM-STING in eukaryotes, and we agree with the reviewer that this is unlikely. Although we do not have sufficient data to speak to the origin of the STING domain itself, we do feel confident in our evidence of domain shuffling. Our illustration in Fig 5B was meant to correspond to the following statement: “Drawing on a shared ancient repertoire of protein domains that includes STING, TIR, and transmembrane (TM) domains, bacteria and eukaryotes have convergently evolved similar STING proteins through domain shuffling.” We believe this inference valid and best describes our results for STING.*

3. Line 119: While the role of Mab21L1-2 are established for development, I'm unaware of a role for MB21D2 in development (or any other phenotype).

*We agree with the reviewer that MB21D2 has not been shown to have any phenotype and have corrected the wording to clarify this point.*

*The line now reads “However, the immune functions of Mab21L1 and MB21D2 remain unclear, although Mab21L1they has been shown to be important for development*[*[29–31]*](https://paperpile.com/c/6RKR01/gye3+Ksxw+JF53)*.”*

4. Line 210: "Gamma" should be "genes"

*We have corrected this error and replaced the word.*

 Reviewer #3 (Significance (Required)):

 This work is of high quality, is timely, and will have a large impact on shaping the field. The origins and evolution of antiviral immunity from bacteria to eukaryotes have been investigated from multiple angles. While the phylogeny and evolutionary trajectory of these genes have been traced in bacteria, there have been relatively fewer analyses across diverse (non-metazoan) eukaryotes. For this reason, I am confident that this manuscript will help future researchers select homologs for investigation and guide similar analyses of other bacterial defense systems.

 A particular challenge of this work is accounting for gene loss across taxa and weighing that possibility against horizontal gene transfer. The authors are conservative in their conclusions and well-reasoned. The comments I have can be addressed with changes to the writing and emphasis of certain points.

 I expect these findings to be of interest to a broad audience of evolutionary biologists, microbiologists, and immunologists.
